# Supplementary material for: “Survey on the burden, epidemiological and clinical characteristics of snakebite envenoming in the Health Demographic Surveillance System (HDSS) of Taabo (Southern Côte d’Ivoire)”
Source: PLoS Negl Trop Dis. 2025 Apr 7;19(4):e0012983. doi: 10.1371/journal.pntd.0012983 (PMC12002634; doi:10.1371/journal.pntd.0012983)
Supplement: S1 File — (PDF) [file pntd.0012983.s002.pdf]

# SURVEY FORM ON SNAKEBITE ENVENOMING IN THE AHONDO HEALTH AREA (TAABO HDSS - CÔTE D'IVOIRE).

## Section 1: General information for snakebite victims

| N° | QUESTIONS | ANSWERS | JUMP |
|---|---|---|---|
| **Q101** | Date of visit | [__\|__]/[__\|__]/[__\|__] (dd/mm/yyyy) | |
| **Q102** | HDSS ID of the victim's household | [________________________________] | |
| **Q103** | HDSS ID of victim | [________________________________] | |
| **Q104** | Victim's place of residence | 1. Village<br>2. Camp | |
| **Q105** | Is the person bitten by the snake: | 1. Alive, (she answers the questions)<br>2. Died because of the bite, (a relative or other household member answers the questions)<br>**3.** Died from a cause unrelated to the bite (a relative or other household member answers the questions) | |
| **Q106** | Informed consent obtained | 1. Yes<br>2. No | |
| **Q107** | Date of consent obtained | [__\|__]/[__\|__]/[__\|__] (dd/mm/yyyy) | |
| **Q108** | How long ago did the snakebite occur? | 1. A few days ago (how many [__\|__])<br>2. A few weeks ago (how many [_\|__])<br>3. A few months ago (how many [__\|__])<br>4. A few years ago (how many [__\|__])<br>5. I don't remember | |

## Section 2: Socio-demographic and economic information on snakebite victims

| N° | QUESTIONS | ANSWERS | JUMP |
|---|---|---|---|
| **Q201** | Sex of victim | 1. Male<br>2. Female | |
| **Q202** | Age of victim | [__\|__]__](year) | |
| **Q203** | Nationality of victim | 1. Côte d'Ivoire<br>2. Ghana<br>3. Bénin<br>4. Burkina-Faso<br>5. Guinée<br>6. Liberia<br>7. Mali<br>8. Togo<br>9. Other (please specify) :…………… | |
| **Q204** | Victim's level of education | 1. No level<br>2. Preschool<br>3. Primary<br>4. Secondary<br>5. Superior<br>6. Koranic<br>7. Not declared<br>8. Not applicable | |
| **Q205** | Marital status of the victim | 1. Single<br>2. Married (Traditional; Civil and/or religious)<br>3. Divorced<br>4. Widow/widower | |
| **Q206** | Type of victim's household construction | 1. Traditional hut<br>2. Hut<br>3. Common courtyard<br>4. Modern villa<br>5. Simple house<br>6. Terraced house<br>7. Traditional-modern house | |
| **Q207** | Nature of the victim's household wall | 1. Wood<br>2. Sheet metal<br>3. Banco / rammed earth<br>4. Straw<br>5. Geoconcrete<br>6. Brick (Hard) | |
| **Q208** | Nature of the snakebite victim's household floor | 1. Soil or sand<br>2. Cement<br>3. Tiles/marble<br>4. Carpet | |
| **Q209** | Main source of lighting of the victim's household | 1. Spark plug<br>2. Storm lamp<br>3. Electricity (CIE)<br>4. Gas lamp | |

| | | | |
|---|---|---|---|
| | | 5. Solar energy<br>6. Generator | |
| **Q210** | The victim's professional activity | 1. Farmer<br>2. Civil servant<br>3. Private employee<br>4. Artisans<br>5. Pupil/student<br>6. Shopkeeper<br>7. Retired<br>8. Housewife<br>9. Not applicable<br>10. Not declared. | |
| **Q211** | Please indicate the source of your annual income | 1. Agricultural activities<br>2. Other activities | |

## Section 3: Socio-environmental factors associated with snakebites

| N° | QUESTIONS | ANSWERS | JUMP |
|---|---|---|---|
| **Q301** | In what environment did the bite occur? | 1. At home<br>2. At work<br>3. In the field<br>4. In the forest<br>5. In a stream<br>6. On a tree<br>7. On the road<br>8. Other (please specify) :………… | |
| **Q302** | What time of day did the snake bite occur? | 1. At dawn in the morning<br>2. In the morning<br>3. At noon<br>4. Afternoon<br>5. In the evening<br>6. At night | |
| **Q303** | What were you doing when the snake bit you? | 1. Sleep/alit<br>2. Field work<br>3. Walking<br>4. Lunch<br>5. Craft activities<br>6. Leisure activities<br>7. Fishing activities<br>8. Household activities<br>9. Other (please specify).................. | |
| **Q304** | Have you seen the snake? | 1. Yes<br>2. No | *If Yes, ask Q306*<br><br>*If No, go to Q401 in section 4* |

| | | | |
|---|---|---|---|
| **Q305** | If yes, give the name of the snake according to local references | 1. Name of snake: …………………<br>2. I do not know | |

## Section 4: Clinical characteristics and management of snakebite victims

| N° | QUESTIONS | ANSWERS | JUMP |
|---|---|---|---|
| **Q401** | **What happened after the snake bite?** | | |
| **Q401a** | Inability to lift the eyelids of the person bitten by the snake | 1. Yes<br>2. No<br>3. Don't know | |
| **Q401b** | Inability to lift the arms and legs of the person bitten by the snake | 1. Yes<br>2. No<br>3. Don't know | |
| **Q401c** | Inability to raise the head while lying on the floor on one's back | 1. Yes<br>2. No<br>3. Don't know | |
| **Q401d** | Bleeding from the mouth of the person bitten by the snake | 1. Yes<br>2. No<br>3. Don't know | |
| **Q401e** | Bleeding at the site of the snakebite | 1. Yes<br>2. No<br>3. Don't know | |
| **Q401f** | Red urine from a snake bite | 1. Yes<br>2. No<br>3. Don't know | |
| **Q401g** | Swelling at the site of the snakebite | 1. Yes<br>2. No<br>3. Don't know | |
| **Q401h** | Swelling of the whole arm/leg of the person bitten by the snake | 1. Yes<br>2. No<br>3. Don't know | |
| **Q401i** | Did the person lose consciousness shortly after the snakebite? | 1. Yes<br>2. No<br>3. Don't know | |
| **Q402** | **What was done after the snake bite?** | | |
| **Q402a** | Was a tourniquet applied? | 1. Yes<br>2. No<br>3. Don't know | |
| **Q402b** | Has the skin been cut where the snakebite occurred? | 1. Yes<br>2. No<br>3. Don't know | |
| **Q402c** | Was a ‘black stone’ applied to the site of the bite? | 1. Yes<br>2. No<br>3. Don't know | |
| **Q403** | **Where did you first turn for help after the snake bite?** | | |

| | | | |
|---|---|---|---|
| **Q403a** | ***At the (village or district) hospital?*** | 1. Yes 2. No | *If Yes, ask questions Q403b to Q403g*<br><br>*If No, go to Q403h* |
| **Q403b** | If yes, did you receive anti-venom in hospital? | 1. Yes 2. No | |
| **Q403c** | Did you receive a blood transfusion in hospital? | 1. Yes 2. No | |
| **Q403d** | Did you receive respiratory assistance in hospital? | 1. Yes 2. No | |
| **Q403e** | Have you been vaccinated against tetanus? | 1. Yes 2. No | |
| **Q403f** | Did you receive wound care for longer because a large wound developed after the snakebite? | 1. Yes 2. No | |
| **Q403g** | If yes, how long have you been in hospital? | 1. A few hours ago (How many [__\|__])<br>2. A few days ago (How many [__\|__])<br>3. A few weeks ago (How many [__\|__]) | |
| **Q403h** | ***Traditional self-medication ?*** | 1. Yes 2 . No | *If yes, ask Q403i*<br><br>*If No, go to Q403j* |
| **Q403i** | If yes, specify traditional self-medication | ………………………………………… | |
| **Q403j** | ***Self-medication with medical products?*** | 1. Yes 2 . No | If Yes, ask Q403k and Q403l<br><br>If No, go to Q403m |
| **Q403k** | If yes, specify the medical products | ……………………………………… | |
| **Q403l** | Where do you buy medical products? | 1. Hospital pharmacy<br>2. Private pharmacy<br>3. Street vendors<br>4. Market vendors<br>5. Other (please specify): ........ | |
| **Q403m** | ***Treated by traditional healers*** | 1. Yes 2 . No | *If Yes, ask Q403n*<br><br>*If No, go to Q404a* |
| **Q403n** | If Yes, specify the treatment of the traditional healer | ……………………………………… | |
| **Q404** | **What happened after you returned home?** | | |
| **Q404a** | Have you had any 'acute problems' at home linked to snakebite? | 1. Yes<br>2. No<br>3. Don't know | *If Yes, ask Q404b*<br><br>*If No, go to Q404c* |
| **Q404b** | If yes, specify the 'acute problems' encountered | ……………………………………….. | |
| **Q404c** | Do you have any 'chronic problems' linked to snakebite? | 1. Yes<br>2. No<br>3. Don't know | *If Yes, ask Q404d*<br><br>*If No, go to Q405* |

| | | | |
|---|---|---|---|
| **Q404d** | If yes, specify the 'chronic problems' encountered | ……………………………………….. | |
| **Q405** | Locating the snake bite on the victim's body | 1. Head<br>2. Neck<br>3. Arm<br>4. Forearm<br>5. Hands<br>6. Fingers<br>7. Chest<br>8. Belly<br>9. Back<br>10. Thigh<br>11. Leg<br>12. Feet<br>13. Toes<br>14. Other (please specify):.......... | |
| **Q406** | Take a photo (of the wound or snakebite scar) | 1. Yes<br>2. No | |
