# Supplementary material for: “Survey on the burden, epidemiological and clinical characteristics of snakebite envenoming in the Health Demographic Surveillance System (HDSS) of Taabo (Southern Côte d’Ivoire)”
Source: PLoS Negl Trop Dis. 2025 Apr 7;19(4):e0012983. doi: 10.1371/journal.pntd.0012983 (PMC12002634; doi:10.1371/journal.pntd.0012983)
Supplement: Attachment — Submitted filename: RESPONSE-TO-REVIEWERS_PNTD-D-25-00039.pdf [file pntd.0012983.s004.pdf]

CSRS
Centre Suisse de Recherches
Scientifiques en Côte d`Ivoire

Abidjan, 24 February 2025

RE: Submission of the revised manuscript to PLoS Neglected Tropical Diseases: (PNTD-D-25-00039)

Dear Editors

We thank you and the reviewers very much for the comments and suggestions. Please find attached the response to the review and the revised version of the manuscript in a track-changes and a clean version.

Following the suggestion of one of the reviewers, we changed the title of the manuscript slightly to ***"Survey on the burden, epidemiological and clinical characteristics of snakebite envenoming in the Health Demographic Surveillance System (HDSS) of Taabo (Southern Côte d'Ivoire)"***

We sincerely hope that the revisions will meet your expectations.

We look forward to hearing from you and thank you for your interest in our study.

Sincerely

Dr Tanoh N'KRUMAH

(On behalf of the co-authors)

'Response to Review'

Methods

Reviewer #2: The sampling technique does not seem at all clear or detailed.
I didn't see the sample size calculation anywhere. This has negative repercussions, as the small number of people who have experienced snakebite and venom does not provide adequate power to generalize the results.
In view of the small number of 11 people bitten by the snake over the study period, non-parametric statistics were indicated, but this is not the case for what the authors were able to do.

Response 1: The sampling technique used in our study is exhaustive sampling within the well-defined framework of a Health and Demographic Surveyance System (HDSS).

We capitalize on the HDSS infrastructure where demographic, health, socioeconomic, environmental, etc. data routinely collected to analyze snakebite envenoming in its context.

Despite the small population we survey, we could clearly demonstrate in this first snakebite study in Taabo HDSS that snakebite is a problem in the Taabo HDSS area and that it is unattended.

The HDSS framework provides the opportunity to continue prospective data collection on snakebites as a new component of the grand HDSS surveys conducted at half-yearly intervals and record changes over time. The snakebite study can be expanded into the entire HDSS population of 78.000 people.

To make our sampling strategy clearer, we added "exhausting sampling" and explained the framework of the sampling within the existing HDSS more fully (**Line 188 to 191**).

Can you justify the choice of design for this study????

Response 2: Please see **Response 1**.

The article says nothing about how the sample size was calculated and nothing about the sampling technique used to select the household.

Response 3: Please see **Response 1**.

Results

Reviewer #2: The annual snakebite incidence per 100,000 population calculated from group 1 was 280.3 per 100,000 (CI: 140.0 - 501.0). The AHA snakebite case-fatality rate calculated from group 2 was 2.0% (95% CI: 0.05 - 10.5) (Table 2).

Can you explain these very wide confidence intervals?

Response 4: The very wide confidence interval of the snakebite incidence and case fatality rate is explained by the fact that we surveyed a small population of just under 4,000 people and recorded a small number of snakebites for group 1 from which we calculated this incidence. We have addressed the limitation of the small population size surveyed and plan to expand the study into the entire population of the Taabo HDSS of 78,000). Please also **see Response 1**.

Although the confidence interval of the snakebite incidence and case fatality rate is wide, our results are within the range of the previous studies carried out in Côte d'Ivoire.

Despite the small population we survey, we could clearly demonstrate in this first snakebite study in Taabo HDSS that snakebite is a problem in the Taabo HDSS area and that it is unattended.

What is the relevance of the choice of these two comparison groups?

Response 5: The two groups are not comparison groups. We divided into two groups to minimize recall bias in the calculation of snakebite incidence which we calculated from Group 1 comprising snakebites experienced in the 12 months prior to the survey (2 October 2022 and 2 October 2023)

In Group 2 we included all individuals experiencing a snakebite at any time before 2 October 2022 to obtain a broader view of the snakebite problem in the Taabo HDSS, while accepting a greater recall bias in group 2 compared to group 1.

We present the two groups in the Tables to keep the snakebite reports with different recall bias separated. We felt that applying statistics to the two groups is overstretching the analysis which – in our view - should be mostly descriptive given the sample size, the incidence of snakebites and the recall bias.

It is, however, reassuring that by and large the results did not differ substantially between the two groups, i.e. the two data collection periods, despite the potentially differing recall bias implied.

The annual incidence of snakebites was significantly higher in seasonal agricultural camps (3296.7 cases per 100,000 inhabitants) than in villages (208.7 cases per 100,000 inhabitants). Wouldn't this result be different if the size were larger?

Response 6: If the sample size was larger, we think that the incidence of snakebites in the camps would still be higher than in the village town.

This is because the camps are generally located far from the villages, in purely agricultural areas, where snakes are most prevalent. The habitats of the people living in the camps offer more refuge and prey for snakes than those in the villages, thus increasing the risk of encounters between the people living in the camps and snakes. In addition, the people living in the camps are permanently involved in agricultural or harvesting activities and, thus, permanently exposed to snakebites.

Have you tried a non-parametric multivariate model with incidence as the dependent variable, given that univariate analyses are not reassuring?

Response 7: As mentioned in **Response 4**, the annual incidence of snakebites in our study was calculated using cases from group 1 (11 cases) to minimize recall bias. These data were not appropriate for the implementation of a multivariate model in this first snakebite study, the aim of which was to describe the epidemiological and clinical characteristics of snakebites in the Taabo HDSS. We mentioned this limitation. Regarding the expansion of the survey, please **see Response 1**.

Snakebite occurs most often in the morning and at night. Have you used a statistical test?

Response 8: Please, see **Responses 5 and 7**.

Reviewer #3: Can't really assess if the analysis matches the analysis plan. The results are logically presented. The sample size justification is missing.

Please, see **Response 1**.

Conclusion

Reviewer #2: The authors do not explain how this data, which lacks power, can be used and generalized to advance knowledge.

Response 9: Although the number of snakebite envenoming cases is low, our results are in line with the data from other parts of West Africa, adding further data and highlighting the importance of snakebite envenoming for this part of the region. (see line 407 - 410 and line 415 - 417).

Editorial and Data Presentation Modifications?

Reviewer #3: Some minor revisions could be using consistent terminology for envenoming. There is some interchanging of envenomation and envenoming. Stick to one term.

Also the discussion section needs a bit of reordering because it seems to jump about. Consider reordering for smoother flow.

Response 10: Corrected to the use of 'envenoming' throughout the article

Summary and General Comments

Reviewer #2: The authors need to improve the writing on the sampling technique and improve the power through a sample that allows us to generalize.
Parametric statistical tests are not appropriate given the small size of the subjects who suffered bites.
The univariate analyses alone do not seem to us to be at all robust for a good generalization.

Response 11: Please see **Responses 1, 5 and 7.**

Reviewer #4: Introduction

Line 87: Delete a space after in 2017

Correction made in **line 88**.

Lines 97-102: It is important here to specify whether these statistics concern the world or a given region

Correction made in **line 98**

Line 107: Please specify the scientific name of the viper species in question, in particular Echis carinatus

Correction made in **line 109**

Line 114 delete "- ' after 'national Household

Correction made in **line 116**

Line 114-119: Here, you discuss national data for Côte d'Ivoire on snakebite incidence and mortality, based on a source from the 200th century, whereas more recent figures have been produced by studies by Habib et al. (2015). Why did you choose this option?

Response 12: We selected here the paper of Chippaux (2002) because it provides information on the epidemiology of snakebites in the Tiassalé health district, where Taabo HDSS is located.

Line 147 study area
It's important here to better present or explain HDSS. Is HDSS a digital data collection platform or a research station? I read that "Le Système de Surveillance Sanitaire et Démographique de Taabo is a research station of the Centre Suisse de Recherches Scientifiques en Côte d'Ivoire (CSRS) located in south-central Côte d'Ivoire, about 150 km northwest of Abidjan." If this is the case, I think that the title of the manuscript needs to be revised, in particular to read: "based on HDSS". In any case, it is important to better explain the content of the HDSS.

Changes made in **line 151 to 159** in the study area section

Proposed new article title (see **line 7 to 9**)

"Survey on the burden, epidemiological and clinical characteristics of snakebite envenoming in the Health Demographic Surveillance System (HDSS) of Taabo (Southern Côte d'Ivoire)"

Results
Line 311-313: Why did none of the victims receive antivenom when this is the main treatment recommended in hospitals? It would be important to mention the reason for this.
Line 314-315. It would be important to identify and mention the scientific names of the snakes responsible for the envenomations.

Response 13: Addressed and text added in **line 320 to 325**

Discussion
Line 409: Delete "[".

Correction made in **line 410**.

Line 410-411: Complete this sentence with : "and are therefore not overly involved in the rural activities that are the main source of snakebites."

Correction made (**line 412 to 414**).

Line 455: Please specify the scientific name of the spitting cobra."

Change made in **line 458**.
